# Revisiting the Hybridization Processes in the *Triatoma brasiliensis* Complex (Hemiptera, Triatominae): Reproductive Isolation between *Triatoma petrocchiae* and *T. b. brasiliensis* and *T. lenti*

**DOI:** 10.3390/insects12111015

**Published:** 2021-11-11

**Authors:** Luiza Maria Grzyb Delgado, Jader de Oliveira, Amanda Ravazi, Fernanda Fernandez Madeira, Yago Visinho dos Reis, Heloisa Pinotti, Ana Beatriz Bortolozo de Oliveira, Isabella da Silva Masarin, Maurício Lilioso, Elaine Folly-Ramos, Cleber Galvão, Maria Tercília Vilela de Azeredo-Oliveira, João Aristeu da Rosa, Kaio Cesar Chaboli Alevi

**Affiliations:** 1 Instituto de Biociências, Universidade Estadual Paulista “Júlio de Mesquita Filho” (UNESP), Rua Dr. Antônio Celso Wagner Zanin, 250, Distrito de Rubião Júnior, Botucatu 18618-689, Brazil; lu.grzyb@gmail.com (L.M.G.D.); amandaravazi95@gmail.com (A.R.); yagoreis@outlook.com.br (Y.V.d.R.); isabella.masarin@gmail.com (I.d.S.M.); kaiochaboli@hotmail.com (K.C.C.A.); 2 Laboratório de Entomologia em Saúde Pública, Departamento de Epidemiologia, Faculdade de Saúde Pública, Universidade de São Paulo (USP), Av. Dr. Arnaldo 715, São Paulo 01246-904, Brazil; jdr.oliveira@hotmail.com; 3 Laboratório de Parasitologia, Departamento de Ciências Biológicas, Universidade Estadual Paulista “Júlio de Mesquita Filho” (UNESP), Faculdade de Ciências Farmacêuticas, Câmpus de Araraquara, Rod. Araraquara-Jaú km 1, Araraquara 14801-902, Brazil; helopinotti@hotmail.com (H.P.); joaoaristeu@gmail.com (J.A.d.R.); 4 Laboratório de Biologia Celular, Departamento de Biologia, Instituto de Biociências, Letras e Ciências Exatas, Câmpus de São José do Rio Preto, Universidade Estadual Paulista “Júlio de Mesquita Filho” (UNESP), Rua Cristóvão Colombo 2265, São José do Rio Preto 15054-000, Brazil; fernanda.bio56@hotmail.com (F.F.M.); anabbortolozo@gmail.com (A.B.B.d.O.); tercilia.vilela@unesp.br (M.T.V.d.A.-O.); 5 Instituto de Biologia, Universidade Estadual de Campinas (UNICAMP), Rua Monteiro Lobato, 255, Campinas 13083-862, Brazil; mauriciolilioso@hotmail.com; 6 Centro de Ciências Aplicadas e Educação, Laboratório de Ecologia Animal, Departamento de Engenharia e Meio Ambiente, Universidade Federal de Paraíba (UFPB), Av. Santa Elizabete, 160, Rio Tinto, Paraíba 58297-000, Brazil; elafolly@yahoo.com.br; 7 Laboratório Nacional e Internacional de Referência em Taxonomia de Triatomíneos, Instituto Oswaldo Cruz (IOC/FIOCRUZ), Av. Brasil 4365, Pavilhão Rocha Lima, Sala 505, Rio de Janeiro 21040-360, Brazil

**Keywords:** triatomines, reproductive barriers, hybridization, speciation, Chagas disease vectors

## Abstract

**Simple Summary:**

Although all triatomines are potential vectors of Chagas disease, there are species with greater or lesser vectorial importance. Therefore, the correct identification of triatomine species is essential for vector control programs. In general, triatomines are identified by external morphological characters. However, some species are very similar or even morphologically identical, making the use of complementary analyses for the correct identification of species important. For this reason, this study focused on the use of experimental crosses to assess the specific status of species of the *Triatoma brasiliensis* species complex. The crosses did not result in hybrids, demonstrating that there are pre-zygotic reproductive barriers installed between *T. petrocchiae* and the other species of the *T. brasiliensis* complex (which confirms the specific status of the species according to the biological species concept). On the basis of the results above, we demonstrated that *T. petrocchiae* is reproductively isolated from *T. b. brasiliensis* and *T. lenti*. Furthermore, we suggest that *T. petrocchiae* is the species most derived from the *T. brasiliensis* complex.

**Abstract:**

*Triatoma petrocchiae* is a species morphologically similar to *T. b. brasiliensis* (which resulted in a synonymization event); despite this similarity, genetic, morphological, and experimental crossbreeding studies confirmed the specific status of *T. petrocchiae*. Considering that both species have been reported living in sympatry and that, for a long time, most species of the *T. brasiliensis* complex were considered only chromatic variants of *T. b. brasiliensis*, we carried out experimental crosses between *T. b. brasiliensis* and *T. petrocchiae* (to confirm whether these species are reproductively isolated) and between *T. lenti* and *T. petrocchiae* (to assess whether *T. petrocchiae* also presents prezygotic isolation with the other species of the *T. brasiliensis* complex). Reciprocal experimental crosses were conducted, and weekly, the eggs were collected, counted, and separated in new containers to assess the hatch rate. Neither cross resulted in hybrids, demonstrating that there are pre-zygotic reproductive barriers installed between *T. petrocchiae* and the other species of the *T. brasiliensis* complex. On the basis of the results above, we demonstrated that *T. petrocchiae* is reproductively isolated from *T. b. brasiliensis* and *T. lenti*. Furthermore, we suggest that *T. petrocchiae* is the species most derived from the *T. brasiliensis* complex.

## 1. Introduction

Triatomines (Hemiptera, Triatominae) are hematophagous insects of great importance to public health, as they are considered the main form of transmission of the protozoan *Trypanosoma cruzi* (Chagas, 1909) (Kinetoplastida, Trypanosomatidae), the etiological agent of Chagas disease [1] (a neglected disease that affects about 8 million people and results in 10,000 deaths per year [1]). There are currently 157 species grouped into 18 genera and five tribes [2,3,4,5]. The Triatomini and Rhodniini tribes have the largest number of species (115 and 24, respectively) and are the most important from an epidemiological point of view (since *Panstrongylus megistus* (Burmeister, 1835), *Triatoma infestans* Klug, 1834, *T. brasiliensis brasiliensis* Neiva, 1911, *T. dimidiata* (Latreille, 1811), and *Rhodnius prolixus* Stål, 1859 are of worldwide importance in the transmission of the disease [6]).

The genus *Triatoma* Laporte, 1832 is the most representative (81 species) and the most morphologically diversified [6,7]. This genus is paraphyletic [8,9], and species are grouped into complexes and subcomplexes [9,10,11,12]. The *T. brasiliensis* complex is a grouping of endemic species from Brazil [13] composed of six species and two subspecies that share a common ancestry: *T. b. brasiliensis* Neiva, 1911, *T. b. macromelasoma* Galvão, 1965, *T. juazeirensis* Costa and Félix, 2007, *T. sherlocki* Papa et al., 2002, *T. petrocchiae* Pinto and Barreto, 1925, *T. lenti* Sherlock and Serafim, 1967, *T. bahiensis* Sherlock and Serafim, 1967, and *T. melanica* Neiva and Lent, 1941 [14,15,16,17,18]. The last taxon grouped in this complex was *T. petrocchiae* [18], a species reported in the states of Bahia, Ceará, Pernambuco, Paraíba, and Rio Grande do Norte [13,19]; however, the potential distribution map published by Caranha et al. [20] suggests that this species could also be found in the states of Piauí, Alagoas, and Sergipe, where the species has not been recorded to date.

The species *T. brasiliensis sensu stricto* is currently divided into two subspecies (*T. b. brasiliensis* and *T. b. macromelasoma*), which can be differentiated by morphological characters: *T. b. brasiliensis* presents a pronotum with 1 + 1 brownish-yellow areas extending from the posterior portion of the anterior lobe to the posterior lobe, femora with broad brownish-yellow rings, and membrane of hemelytra with a lumen of cells that are not darkened; *T. b. macromelasoma* presents a pronotum with 1 + 1 narrow brownish-yellow stripes on the submedian carinae, not attaining its apex, legs with an incomplete brownish-yellow ring on the apical half of the femora, and hemelytra with membrane cells that are darkened on the central portion [21]. In addition to the phenotypic divergences, these species have a different geographic distribution: while *T. b. brasiliensis* has been noted in the states of Ceará, Maranhão, Paraíba, Piauí, and Rio Grande do Norte, *T. b. macromelasoma* is endemic to Pernambuco [13,19].

*Triatoma petrocchiae* is a species morphologically similar to *T. b. brasiliensis*, which led Lucena [22] to propose the synonymization of species, considering *T. petrocchiae* only as a chromatic variant of *T. b. brasiliensis*. However, Espínola [23] carried out experimental crosses between *T. b. brasiliensis* and *T. petrocchiae* from Paulo Afonso, Bahia, Brazil, and observed that these species did not produce viable hybrids. On the basis of this, Lent and Wygodzinsky [24] revalidated the specific status of *T. petrocchiae* from morphological data (the status was corroborated with genetic analyses using allozyme electrophoresis [25]).

The interspecific crosses performed by Espínola [23] were proposed because chromatic variations were observed in the populations of *T. b. brasiliensis* from Paulo Afonso, Bahia. The authors indicated that there are similarities in the coloration between *T. b. brasiliensis* and *T. petrocchiae* and, above all, these species share the same ecological niche. Considering that these species have been reported living in sympatry [26,27] and that, in 1971, most species of the *T. brasiliensis* complex were still considered only chromatic variants of *T. b. brasiliensis*, there is a need to confirm whether *T. b. brasiliensis* and *T. petrocchiae* are really reproductively isolated (mainly because all other species in this complex are capable of producing hybrids [15,28,29,30,31,32]). On the basis of the assumptions above, we carried out experimental crosses between *T. b. brasiliensis* and *T. petrocchiae* (to corroborate the results of Espínola [23]) and between *T. lenti* and *T. petrocchiae* (to assess whether *T. petrocchiae* also presents prezygotic isolation with the other species of the *T. brasiliensis* complex).

## 2. Materials and Methods

**Figure 1:**
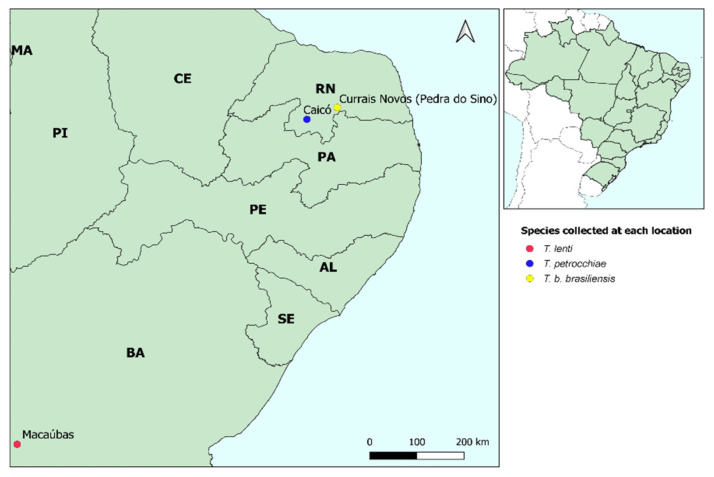
Distribution map of species used in experimental crosses.

Reciprocal experimental crosses were conducted between *T. b. brasiliensis* (from Currais Novos (Pedra do Sino), Rio Grande do Norte, Brazil, collected in wild ecotopes (geographic coordinates: 6°17′06.8″ S 36°29′51.9″ W)) and *T. petrocchiae* (from Caicó, Rio Grande do Norte, Brazil, collected in wild ecotopes (geographic coordinates: 6°27′47.8″ S 37°09′11.3″ W)) and between *T. lenti* (from Macaúbas, Bahia, Brazil, collected in peridomiciliary ecotopes (geographic coordinates: 13°11′25.7″ S 42°31′56.3″ W)) and *T. petrocchiae* (Figure 1). The insects used in the experiment came from colonies kept in the Triatominae insectary of the School of Pharmaceutical Sciences, São Paulo State University (UNESP), Araraquara, São Paulo, Brazil. The experimental crosses were conducted in the Triatominae insectary, according to the experiments of Mendonça et al. [30], Neves et al. [33], and Pinotti et al. [32]: the insects were sexed as 5th instar nymphs [34], and males and females were kept separately until they reached the adult stage to guarantee the virginity of the insects used in the crosses. For the experimental crosses, three couples from each set were placed in plastic jars (diameter 5 cm × height 10 cm) (each couple in a jar) and kept at room temperature (average of 24 °C [35]) and an average relative humidity of 63% [35]). Weekly, the couples were fed on duck blood, and the eggs were collected, counted, and separated into new containers to assess the hatch rate.

## 3. Results and Discussion

The experimental crosses between *T. petrocchiae* and *T. b. brasiliensis* did not result in hybrids (Table 1), demonstrating that there are pre-zygotic reproductive barriers installed between these species (confirming the specific status of *T. petrocchiae* according to the biological species concept [36,37]). These results obtained for the cross between *T. b. brasiliensis* and *T. petrocchiae* from Rio Grande do Sul (the state where the species were also collected in the same rock outcrop spot [38]) agree with those obtained by Espínola [23] when they crossed specimens from Paulo Afonso, Bahia.

**Figure 2:**
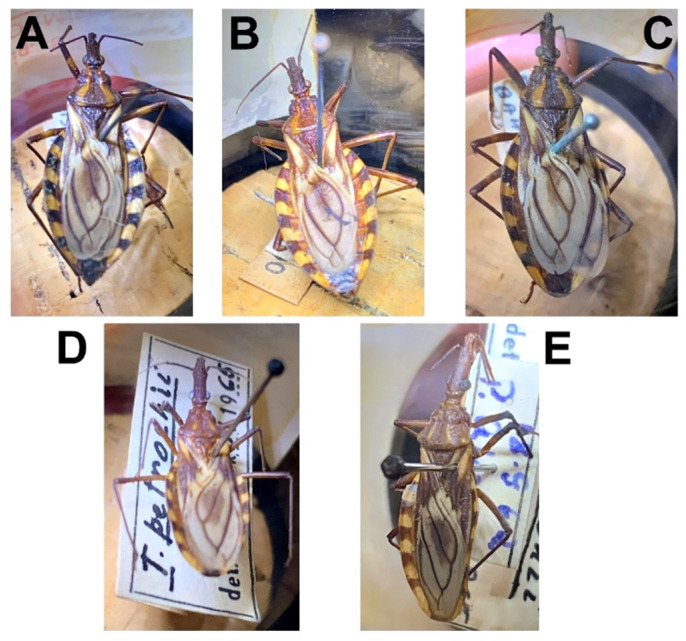
Triatomines deposited in the entomologic collections of the Faculty of Public Health of the University of Sao Paulo, Brazil. (**A**,**B**) *T. b. brasiliensis* ♀ (Brazil, Paulo Afonso, BA. Ident. Sherlock/68); (**C**) *T. b. brasiliensis* ♂ (Brazil, Paulo Afonso, BA. Ident. Sherlock/68); (**D**,**E**) *T. petrocchiae* ♂ (Brazil, Paulo Afonso, BA. Ident. Sherlock/62).

Whereas in 1971, the current species *T. melanica*, *T. sherlocki*, and *T. juazeirensis* were considered only phenotypic variants and/or subspecies of *T. b. brasiliensis* [14,39,40,41,42], we conducted a survey of the literature on the triatomine already noted in Paulo Afonso, Bahia to confirm which species Espínola [22] had crossed with *T. petrocchiae*, and we observed that only *T. b. brasiliensis* and *T. petrocchiae* were the species of the *T. brasiliensis* complex notified for the municipality [43]. Furthermore, to ensure that the *T. brasiliensis* complex triatomines collected in Paulo Afonso, Bahia, were correctly identified as *T. b. brasiliensis* and *T. petrocchiae*, we evaluated some specimens collected in this municipality that were deposited in the entomologic collections of the Faculty of Public Health of the University of Sao Paulo, Brazil (Figure 2). On the basis of this information, we confirmed that the specimens used in the experiments of Espínola [23] were *T. b. brasiliensis*.

Although Espínola [23] suggested that *T. petrocchiae* and *T. b. brasiliensis* share the same ecological niche, Liloso et al. [27] recently demonstrated that while *T. b. brasiliensis* is mainly associated with rodents, the food sources of *T. petrocchiae* were strongly associated with reptiles of the *Tropidurus* and *Hemidactylus* genera; this suggests that *T. petrocchiae* is the single member within this complex that is associated with reptiles, indicating a distinct niche occupation related to the trophic resources. These results point to the possible presence of a prezygotic reproductive barrier due to ecological isolation between *T. petrocchiae* and members of the *T. brasiliensis* complex. However, other possible prezygotic barriers cannot be ruled out, such as mechanical isolation, as the morphological analysis of the external female genitalia evidenced some unique characteristics for *T. petrocchiae* [44].

Experimental crosses between *T. petrocchiae* and *T. lenti* also did not result in hybrids (Table 1). Unlike *T. b. brasiliensis*, which cohabits rock outcrops with *T. petrocchiae* [27,38], there are no reports of *T. petrocchiae* and *T. lenti* living in sympatry (on the contrary, they inhabit municipalities in the state of Bahia that are at least 800 km away [43]). This result is in accordance with what was proposed by Oliveira et al. [18], which suggests that *T. petrocchiae* is the most distant species from the *T. brasiliensis* complex. The genomic incompatibility resulting in the inability to produce hybrids with *T. lenti* points to the hypothesis that *T. petrocchiae* was possibly the first species to be derived from the common ancestor of the *T. brasiliensis* complex (since all other species in the complex produce hybrids [15,28,29,30,31,32]).

The prezygotic isolation observed between *T. petrocchiae* and species of the *T. brasiliensis* complex was only observed when members of this complex (*T. b. brasiliensis*) were crossed with other subcomplexes, such as *T. sordida* [45], *T. infestans* [45], and *T. vitticeps* subcomplexes [33] (which are species phylogenetically distant from *T. b. brasiliensis* [8,9]). The reproductive barrier that possibly prevents hybrids between other species of the *T. brasiliensis* complex is based on post-zygotic reproductive isolation due to hybrid collapse (as noted by Mendonça et al. [30] and Alevi et al. [31]). It was believed that *T. melanica* was the most differentiated form of the complex [37]; however, according to the results of experimental crosses and the high genetic distance observed between *T. petrocchiae* and members of the *T. brasiliensis* complex [46], the most differentiated species from a genetic point of view is *T. petrocchiae*.

Before concluding, it is worth mentioning that the low number of eggs produced by crosses can be a limiting factor for the research. Furthermore, it is important that new crosses between *T. petrocchiae* and all members of the *T. brasiliensis* complex be carried out to confirm that this species is indeed reproductively isolated from all other species in the complex or whether prezygotic isolation is restricted to *T. b. brasiliensis* and *T. lenti* (as noted by Espínola et al. [23] and in the present manuscript).

## 4. Conclusions

On the basis of the results above, we demonstrated that *T. petrocchiae* is reproductively isolated from *T. b. brasiliensis* and *T. lenti* (confirming the specific status of *T. petrocchiae*). Furthermore, we demonstrated that these species have prezygotic reproductive isolation and suggest that *T. petrocchiae* is the species most derived from the *T. brasiliensis* complex.

## Figures and Tables

**Table 1:** Experimental crosses performed between *T. petrocchiae* x *T. b. brasiliensis* and *T. lenti*.

Crossing Experiments					Number of Eggs				Egg Fertility
					C1	C2	C2	Total	
♀	*T. b. brasiliensis*	x	*T. petrocchiae*	♂	48	40	56	144	0%
♀	*T. petrocchiae*	x	*T. b. brasiliensis*	♂	38	45	37	120	0%
♀	*T. lenti*	x	*T. petrocchiae*	♂	42	33	27	102	0%
♀	*T. petrocchiae*	x	*T. lenti*	♂	36	28	22	86	0%
**Parental Crossings**									
♀	*T. b. brasiliensis*	x	*T. b. brasiliensis*	♂	-	-	-	414	95,4%
♀	*T. petrocchiae*	x	*T. petrocchiae*	♂	-	-	-	58	86,2%
♀	*T. lenti*	x	*T. lenti*	♂	-	-	-	179	57,5%

## Data Availability

All relevant data are within the manuscript.
